# Autophagy Facilitates the Development of Breast Cancer Resistance to the Anti-HER2 Monoclonal Antibody Trastuzumab

**DOI:** 10.1371/journal.pone.0006251

**Published:** 2009-07-16

**Authors:** Alejandro Vazquez-Martin, Cristina Oliveras-Ferraros, Javier A. Menendez

**Affiliations:** 1 Catalan Institute of Oncology (ICO), Girona, Catalonia, Spain; 2 Girona Biomedical Research Institute (IdIBGi), Dr. Josep Trueta University Hospital of Girona, Girona, Catalonia, Spain; City of Hope Medical Center, United States of America

## Abstract

Autophagy has been emerging as a novel cytoprotective mechanism to increase tumor cell survival under conditions of metabolic stress and hypoxia as well as to escape chemotherapy-induced cell death. To elucidate whether autophagy might also protect cancer cells from the growth inhibitory effects of targeted therapies, we evaluated the autophagic status of preclinical breast cancer models exhibiting auto-acquired resistance to the anti-HER2 monoclonal antibody trastuzumab (Tzb). We first examined the basal autophagic levels in Tzb-naive SKBR3 cells and in two pools of Tzb-conditioned SKBR3 cells (TzbR), which optimally grow in the presence of Tzb doses as high as 200 µg/ml Tzb. Fluorescence microscopic analyses revealed that the number of punctate LC3 structures -a hallmark of autophagy- was drastically higher in Tzb-refractory cells than in Tzb-sensitive SKBR3 parental cells. Immunoblotting analyses confirmed that the lipidation product of the autophagic conversion of LC3 was accumulated to high levels in TzbR cells. High levels of the LC3 lipidated form in Tzb-refractory cells were accompanied by decreased p62/sequestosome-1 protein expression, a phenomenon characterizing the occurrence of increased autophagic flux. Moreover, increased autophagy was actively used to survive Tzb therapy as TzbR pools were exquisitely sensitive to chemical inhibitors of autophagosomal formation/function. Knockdown of LC3 expression *via* siRNA similarly resulted in reduced TzbR cell proliferation and supra-additively interacted with Tzb to re-sensitize TzbR cells. Sub-groups of Tzb-naive SKBR3 parental cells accumulated LC3 punctate structures and decreased p62 expression after treatment with high-dose Tzb, likely promoting their own resistance. This is the first report showing that HER2-overexpressing breast cancer cells chronically exposed to Tzb exhibit a *bona fide* up-regulation of the autophagic activity that efficiently works to protect breast cancer cells from the growth-inhibitory effects of Tzb. Therapeutic targeting autophagosome formation/function might represent a novel molecular avenue to reduce the emergence of Tzb resistance in HER2-dependent breast carcinomas.

## Introduction

Significant amount of research has been dedicated to elucidate molecular mechanisms that could explain *de novo* and acquired resistance to the anti-HER2 monoclonal antibody trastuzumab (Tzb; Herceptin®), the first immunotherapeutic drug for the successful treatment of breast carcinomas overexpressing the *HER2* (*erb*B-2) oncogene [1]–[8]. Proposed mechanisms for innate or acquired resistance to Tzb include steric inhibition of Tzb binding to the extracellular domain (ECD) of the HER2 tyrosine kinase receptor imposed by other extracellular factors such as the glycoprotein mucin 4 (MUC-4) [9], [10], molecular changes in the target receptor itself (*e.g.* HER2 mutations [11]–[13]; accumulation of a proteolyzed HER2 fragment –p95HER2- lacking the extracellular Tzb binding epitope but retaining ligand-independent TK activity [14]–[16]), and cross-talk with other transduction cascades such as the insulin growth factor (IGF)-1, estrogen receptor (ER) and vascular endothelial growth factor (VEGF) pathways that could compensate for attenuated HER2 signaling [8], [17]–[23]. Alterations in the regulation of HER2 downstream signaling components, including sub-cellular localization of the cyclin-dependent kinase (CDK)-inhibiting protein p27^Kip1^ and independent attenuation of PI-3′K/AKT/mTOR-mediated apoptosis through downregulation of the phosphatase and tensin homolog (PTEN) tumor suppressor have also been implicated as potential sources of resistance to HER2-targeted therapies [24]–[27]. Increased activation of PI-3′K and its downstream effector AKT has also been associated with Tzb resistance in HER2-dependent breast carcinoma cells [28]–[30].

To date, the survival pathway of macroautophagy (also referred as autophagy) has not been implicated in Tzb resistance. Autophagy –lysosomal degradation, or eating (*phagy*), of part of the cell's self (*auto*)- is a catabolic process of organelle digestion that generates ATP during periods on nutrient limitation [31]–[37]. Autophagy optimizes nutrient utilization in rapidly growing cells when faced with hypoxic or metabolic stresses and, hence it contributes to normal and cancer cell survival. During autophagy, macroautophagosomes (also referred as autophagosomes) are formed as double membrane-bound vesicles which engulf cytoplasm and/or cytoplasmic organelles. Then, autophagosomes fuse with lysosomes to degrade the contents of the autophagic vesicle and provide essential building blocks, such as amino acids back to cell. Because this mechanism may be decreased in tumor cells compared with normal cells, initial studies appreciated autophagy as a tumor suppressor mechanism [38], [39]. Indeed, cancer cells may undergo autophagic cell death (APCD; also referred to as active cell death II [ACDII]) following extreme autophagic degradation associated with exposure to several cancer therapies [40]. However, the autophagic response can also function as a protective mechanism allowing the recycling of proteins and cellular components to survive cell injuries induced by cytotoxic agents. Although it is well established that autophagy can protect cancer cells against various stressors, including chemotherapeutics [41]–[44], it remains largely unknown whether “protective autophagy” might also defend cancer cells from the growth inhibitory effects of targeted therapies such as monoclonal antibodies, tyrosine kinase inhibitors, *etc*.

In this study, we investigated the autophagic status in preclinical breast cancer models exhibiting auto-acquired resistance to Tzb that were obtained by continuously growing Tzb-sensitive HER2-overexpressing SKBR3 breast cancer cells in the presence of clinically relevant concentrations of Tzb for more than 10 months. We followed complementary criteria to accurately monitor autophagy in SKBR3-derived Tzb-refractory cells [45]. First, we monitored the level of intermediary structures of the autophagic pathway, *e.g.* the level of endogenous LC3-II/LC3-I (-microtubule-associated protein 1 light chain 3 beta- a specific and sensitive autophagosome marker extensively used to monitor autophagic activity) and the level of p62 protein (also known as SQSTM1 [sequestosome-1]), which serves as a link between LC3 and ubiquitinated substrates destined for autophagic degradation [46]–[54]. Second, to unambiguously establish the pro-survival role of an increased catabolic flux through the autophagic pathway was critical to the development of acquired Tzb resistance, we assessed how chemical autophagy inhibitors or siRNA-induced knockdown of LC3 altered cell proliferation in TzbR cells. Under experimental conditions described here, we report for the first time that induction of autophagy is closely related to the cell survival system acquired by HER2-overexpressing breast cancer cells chronically exposed to Tzb.

## Results

**Figure 1:**
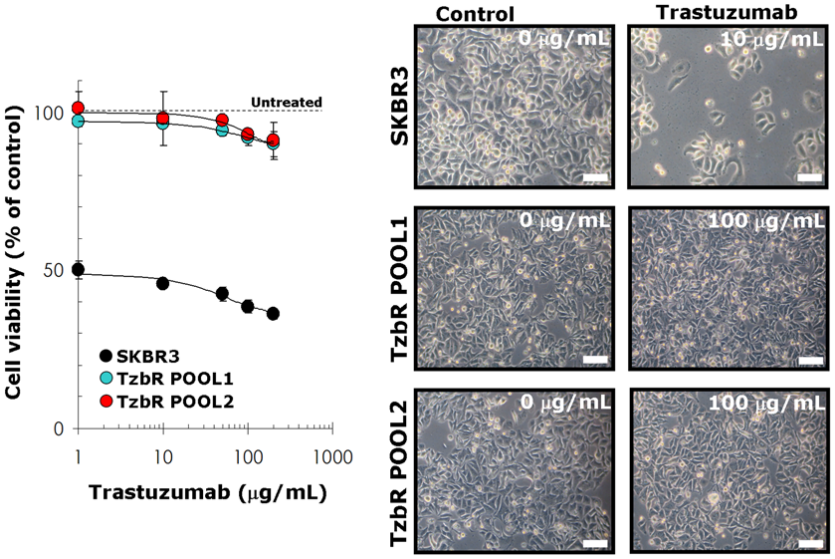
Characterization of Tzb-refractory *HER2*-gene Amplified SKBR3 Breast Cancer Cells. *Left.* The metabolic status of Tzb-naive SKBR3 parental cells and Tzb-refractory TzbR POOLs treated with graded concentrations of Tzb was evaluated using a MTT-based cell viability assays and constructing dose-response graphs as % of untreated cells (dashed line = 100% cell viability). Results are means (*circles*) and 95% confidence intervals (*bars*) of three independent experiments performed in triplicate. Treatment with Tzb concentrations as high as 100 µg/mL Tzb failed to significantly decrease cell viability in TzbR POOLs whereas a 10 times lower concentration of Tzb drastically reduced cell viability (>50%) in SKBR3 parental cells. *Right:* Representative microphotographs of untreated and experimental cell cultures following 72 hours treatment with different concentrations of Tzb, as specified. Examination of microphotographs reveals the smaller size of Tzb-refractory TzbR POOLs when compared to Tzb-naive SKBR3 parental morphology. *Scale bar* = 10 µm.

We established Tzb-resistant HER2-positive breast cancer cells by exposing Tzb-naive SKBR3 parental cells to incremental increases of Tzb. Tzb-resistance selection continued until the SKBR3 cell population could sustain cell viability and proliferate when challenged with 200 µg/ml Tzb. Under these experimental conditions, two pools of Tzb-refractory cells (TzbR POOL1 and TzbR POOL2) were obtained upon exposure of SKBR3 parental cells for a minimum of 10 months before starting any experimental procedure. We confirmed resistance to Tzb by performing MTT-based cell viability assays. When the concentrations of Tzb needed to decrease optical density by 50% were calculated from the percentage of viable cells after exposure to graded concentrations of Tzb, the Inhibitory Concentration 50 (IC_50_) value for SKBR3 parental cells was as low as 2 µg/ml Tzb. Treatment with Tzb at concentrations as high as 200 µg/ml Tzb likewise failed to significantly decrease cell viability in TzbR POOLs (**Figure 1** **, *left panel***). TzbR POOLs exhibited HER2 expression levels comparable or slightly higher than those naturally occurring in SKBR3 parental cells (data not shown).

### Tzb-refractory Cells Exhibit Increased Autophagosome Formation

**Figure 2:**
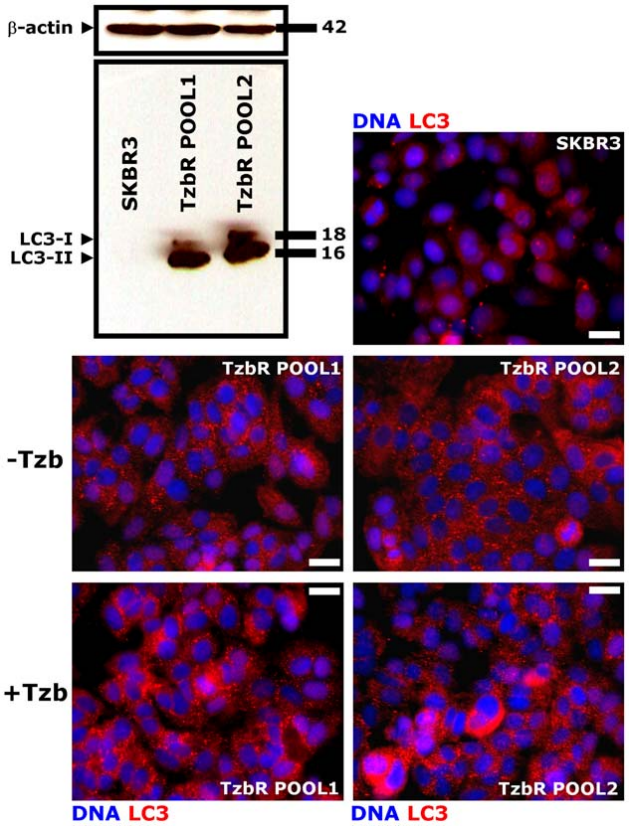
Dynamics of Autophagosome Formation in SKBR3 cells-derived Tzb-Refractory POOLs. *Immunoblotting.* Autophagosome formation in whole cell lysates of Tzb-naive SKBR3 parental cells and Tzb-refractory TzbR POOLs was detected with Western blot analysis using a LC3 antibody. Top band (18 kDa) represents LC3-I. Bottom band (16 kDa) represents LC3-II, a typical marker of autophagosomes. Autophagosome formation is robust in Tzb-refractory TzbR cells when compared to low to undetectable levels of LC3-I/LC3-II expression in Tzb-sensitive SKBR3 parental cells. Figure shows a representative immunoblotting analysis. Equivalent results were obtained in three independent experiments. *Immunofluorescence.* After fixation and permeabilization, cellular distribution of autophagosome marker LC3 was assessed following staining with a LC3 antibody and Hoechst 33258 for nuclear counterstaining. SKBR3 parental cells display a homogenous but weak cytoplasmic LC3 staining, which is typical of absent or low-level autophagosome formation. TzbR POOLs show a marked contrast enhancement in the punctated pattern of endogenous LC3 expression, which is characteristic of autophagosome formation. Of note, Tzb exposure further increases autophagosome LC3 pattern. Images show representative portions of SKBR3 and TzbR cell cultures captured in different channels for LC3 (*red*) and Hoechst 33258 (*blue*) with a 20× objective, and merged on BD Pathway™ 855 Bioimager System using BD Attovision™ software. *Scale bar* = 25 µm.

**Figure 3:**
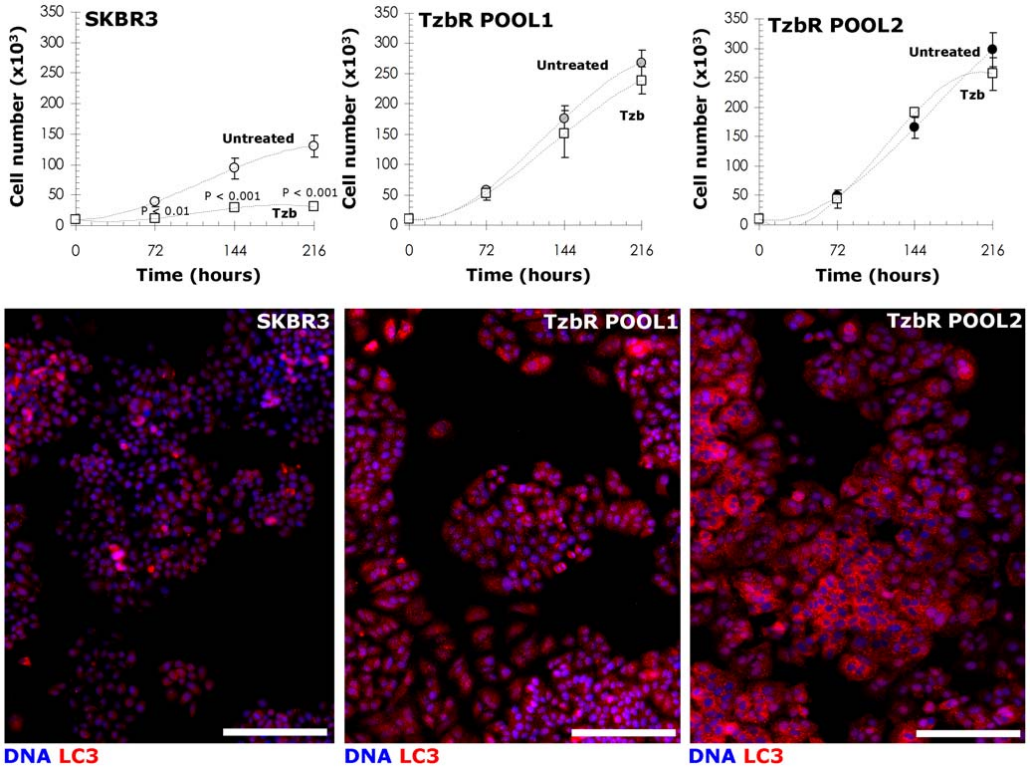
Correlation Between Proliferative Profiles and Dynamics of Autophagosomal Formation in TzbR-refractory Cells. *Top panels.* SKBR3 parental cells and SKBR3-derived Tzb-refractory POOLs were plated in 24-well plates at a density of 10,000 cells/well and cultured with regular medium in the absence or presence of Tzb (10 µg/mL Tzb in SKBR3 parental cells; 100 µg/mL Tzb in TzbR POOLs). The data presented are mean of number cells ×10^5^/well (*circles*) and 95% confidence intervals (*bars*) of three independent experiments made in duplicate obtained after 0, 3, 6 and 9 days. Statistically significant differences (one-factor ANOVA analysis) between experimental conditions and unsupplemented control cells are shown. All statistical tests were two-sided. No statistically significant differences were observed in the number of TzbR cells growing in the presence of 100 µg/mL Tzb up to 9 days whereas 10 µg/mL Tzb significantly reduced cell proliferation in SKBR3 parental cells as early as 3 days after Tzb exposure. Slope of the growth curves clearly denotes a faster proliferation of TzbR POOLs regardless Tzb exposure. *Bottom panels.* Images show representative whole populations of SKBR3 cells and TzbR cells growing in individual wells that were captured using different channels for LC3 (*red*) and Hoechst 33258 (*blue*) as a 4×4 montage with a 20× objective on BD Pathway™ 855 Bioimager System, and merged using BD Attovision™ software. As discussed in Figure 2, TzbR POOLs notably exhibit a marked contrast enhancement in the punctated pattern of endogenous LC3 expression, which is typical for autophagosomal formation. *Scale bar* = 200 µm.

During Tzb selection of the TzbR POOLs, light microscopy examination consistently showed an increase in the number of dark, cytosolic granules in surviving, smaller cells (**Figure 1** **, *right panel***). Because these granules were not related to senescent granules as confirmed by β-galactosidase staining (data not shown), we sought to determine if they may correspond to increased cytosolic levels of autophagosomes. Immunoblotting assessment of LC3 expression is an easy method to predict autophagic activity of mammalian cells because the amount of LC3-II –*i.e.* the product of the autophagic posttranslational modification of LC3- correlates with the number of autophagosomes [45]–[49]. The product of this autophagic conversion of LC3, LC3-II, tightly associates with the autophagosome membrane and migrates faster than LC3-I on SDS-PAGE. Therefore, LC3 immunoblotting may detect two bands: LC3-I with an apparent mobility of 18 kDa and LC3-II (16 kDa). When we utilized this property of LC3 to initially monitor changes in the dynamics of the autophagic process in Tzb-sensitive and in Tzb-refractory cancer cells, both the total amount and, particularly, the lipidation status of LC3 (*i.e.* LC3-I is converted by lipidation to the phosphatidyl-ethanolamine conjugated form LC3-II) were drastically up-regulated in Tzb-unresponsive TzbR POOLs whereas LC3-I/LC3-II proteins were hardly detectable in Tzb-naive SKBR3 parental cells (**Figure 2**). To further confirm that autophagosome formation was increased in Tzb-refractory cells, both the expression and the sub-cellular compartmentalization of LC3 in individual cells and in whole cell cultures were monitored by indirect immunofluorescence using an automated confocal-imaging approach. Untreated SKBR3 parental cells showed a homogenous but weak cytoplasmic staining of LC3, consistent with the distribution of LC3-I and typical of low-level or no autophagosome formation. Consistent with the dramatic increase in LC3 processing assessed by immunoblotting procedures, LC3 localization dramatically changed from diffuse to punctate or dotted pattern in Tzb-refractory TzbR POOLs (**Figure 2**). High content-imaging of whole cell populations growing in individual wells (captured as 4×4 montages) clearly revealed that >90% of TzbR cells exhibited an intense punctate LC3 fluorescence (the medium number of autophagosomes per cell was ∼50) whereas a significantly lower percentage of SKBR3 parental cells (∼5%) revealed LC3-containing vesicles (**Figure 3**). Video confocal microscopy also revealed a robust accumulation of endogenous LC3 in the cytosol of Tzb-refractory cells was, consistent with the distribution of LC3-II-positive bodies representing isolation membranes and autophagosomes (**Supplemental** **Video S1** –SKBR3-, **Supplemental** **Video S2** –TzbR POOL1- and **Supplemental** **Video S3** –TzbR POOL2-). Importantly, this high level of autophagosomes accompanied with healthy-appearing nuclei and high proliferation rates in Tzb-refractory cells.

### Tzb-refractory Cells Exhibit Increased Basal Autophagy

Since the lipidation status of LC3 has been proposed to accurately reflect autophagic activity, our findings revealing an increase in LC3-II expression as assessed by immunoblotting along with an increase in LC3 fluorescent puncta, strongly suggested that augmented autophagosome formation due to increases in autophagic activity may represent a previously unrecognized pro-survival pathway underlying acquired auto-resistance to Tzb. However, autophagy is a dynamic, multi-step process that can be modulated at several steps, both positively and negatively. Therefore, the striking accumulation of autophagosomes as measured by LC3 lipidation on a western blot or fluorescent LC3 dots could also reflect a reduced turnover of autophagosomes in TzbR cells. To determine whether the increase in autophagosome-related LC3 was due to an increase in autophagosome formation or was a secondary consequence of lysosome dysfunction preventing autophagosome clearance in TzbR cells, we initially performed indirect immunofluorescence staining for the lysosomal marker lysosomal-associated membrane protein-1 (LAMP-1). We found that lysosome abundance was increased relative in each Tzb-refractory pool relative to Tzb-naive SKBR3 parental cells (data not shown), thus suggesting that diminished lysosomal function did not contribute to autophagosome accumulation in Tzb-resistant SKBR3 pools. Although these data were consistent with an increase in flux through autophagic clearance pathways, we took additionally two complementary experimental strategies -namely measurement of sequestosome 1 (SQSTM1/p62) protein expression and pharmacological inhibition of autophagy- to further distinguish whether increases in the level of phosphatidylethanolamine-modified LC3 (LC3-II) reflected the induction of autophagy or inhibition of autophagosome clearance upon development of Tzb resistance.

**Figure 4:**
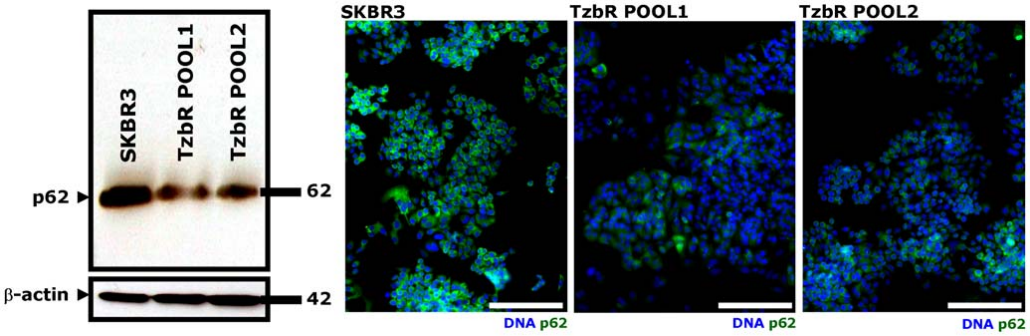
Dynamics of Autophagic Degradation in Tzb-refractory Cells. *Immunoblotting.* Autophagic degradation in whole cell lysates of Tzb-naive SKBR3 parental cells and Tzb-refractory TzbR POOLs was detected with Western blot analysis using a p62 antibody. Immunoblotting bands (64 kDa) represent p62/SQSTM1, a selective substrate of autophagy. Autophagic degradation (*i.e.* down-regulation of endogenous p62 protein expression) is significantly increased in Tzb-refractory TzbR cells when compared to Tzb-sensitive SKBR3 parental cells. Figure shows a representative immunoblotting analysis. Equivalent results were obtained in three independent experiments. *Immunofluorescence.* After fixation and permeabilization, cellular distribution of p62 was assessed following staining with a p62 antibody and Hoechst 33258 for nuclear counterstaining. SKBR3 parental cells display a homogenous and strong cytoplasmic p62 staining, which is typical of absent or low-level of autophagic degradation. TzbR POOLs show a significant decrease in the cytoplasmic distribution of p62 that appears somewhat vesiculated, which is typical of enhanced autophagic degradation. Images show representative whole populations of SKBR3 and TzbR cells growing in individual wells that were captured using different channels for p62 (*green*) and Hoechst 33258 (*blue*) as a 4×4 montage with a 20× objective on BD Pathway™ 855 Bioimager System, and merged using BD Attovision™ software. *Scale bar* = 200 µm.

**Figure 5:**
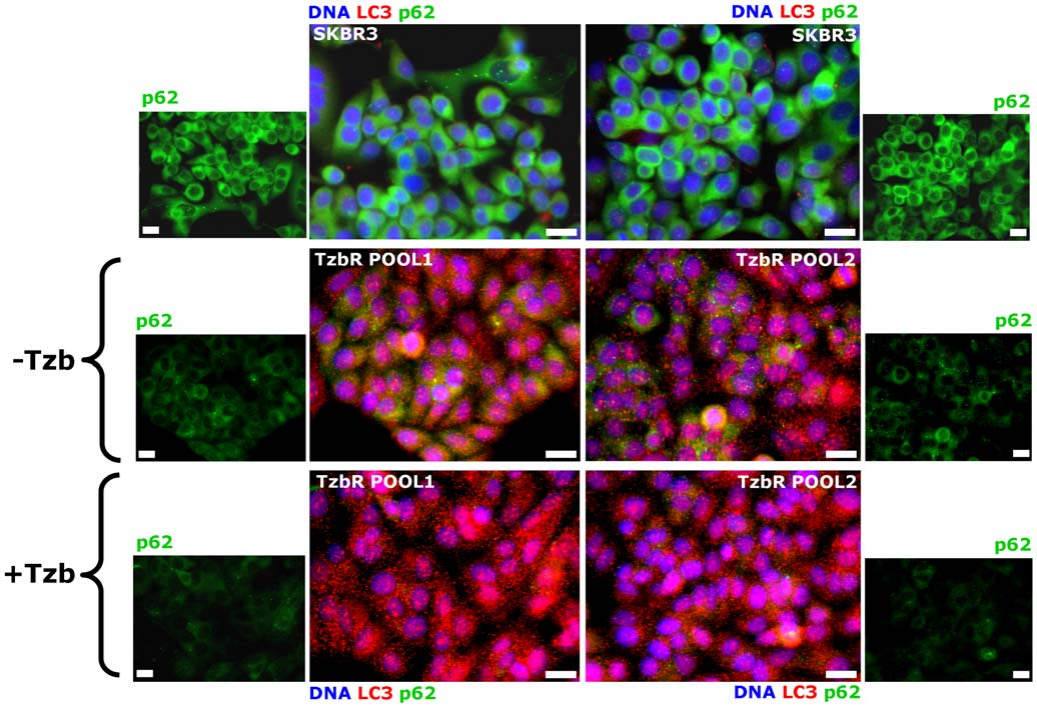
Dynamics of Autophagy Flux in Tzb-refractory cells. Since increased LC3-II levels together with a reduction of p62 protein levels characterize the occurrence of increased autophagic flux, SKBR3 parental cells and TzbR cells (untreated and treated with 100 µg/mL Tzb) were triple stained with antibodies against LC3 and p62 and with Hoechst 33258 for nuclear counterstaining. Tzb-refractory cells exhibit an exacerbated autophagic clearance compared to Tzb-sensitive SKBR3 parental cells when considering overlapping fluorescence of LC3 and p62 as an indirect marker of p62 presenting ubiquitinated protein bodies to the autophagic machinery in a LC3-dependent manner. Images show representative portions of SKBR3 and TzbR cell cultures that were captured using different channels for LC3 (*red*), p62 (*green*) and Hoechst 33258 (*blue*) with a 20× objective and merged on BD Pathway™ 855 Bioimager System using BD Attovision™ software. *Scale bar* = 25 µm.

When high levels of LC3 lipidated form associate with impairment in autophagosome maturation, this phenomenon is accompanied by a marked increase in the level of p62. Conversely, increased LC3-II levels together with a reduction of p62 protein levels characterize the occurrence of a real autophagic flux increase [45], [50]–[54]. The latter scenario was observed in Tzb-refractory TzbR POOLs when compared to Tzb-naive SKBR3 parental cells, thus suggesting that acquired Tzb auto-resistance of Tzb-conditioned cells is accompanied by increased autophagy flux. As shown in **Figure 4**, immunoblotting procedures detected a slight but significant reduction in the total p62 protein content in TzbR-derived whole cell lysates when compared to p62 protein expression status in Tzb-naive SKBR3 parental cells. High content-imaging of whole cell populations growing in individual wells (captured as 4×4 montages) further confirmed that >80% of TzbR cells exhibited a significant decrease in content of p62 per individual cell when compared to Tzb-naive SKBR3 parental cells. Because p62/SQSTM1 is a selective substrate of autophagy and, hence, monitoring endogenous p62 protein levels indirectly measures autophagic degradation, we concurrently monitored how acquisition of breast cancer auto-resistance to Tzb affected cellular distribution of both p62 and LC3 (**Figure 5**). As steady state, the p62 level in Tzb-naive SKBR3 parental cells was much higher than in Tzb-refractory SKBR3 TzbR POOLs. This significant decrease in p62 content per individual cell along with the formation of some punctate vesicular sub-cellular distributions of p62 (p62 bodies) was accompanied by the accumulation of intense punctate LC3 fluorescence in TzbR cells. When comparing overlapping fluorescence of LC3 and p62 -which may be viewed as a good indicative of p62 presenting ubiquitinated protein bodies to the autophagic machinery *via* LC3- it become obvious that Tzb-refractory cells exhibit an exacerbated autophagic clearance compared to Tzb-sensitive SKBR3 parental cells (**Figure 5**).

### Blockade of Macroautophagosome Formation/Function Enhances Tzb Efficacy in Tzb-refractory Cells

**Figure 6:**
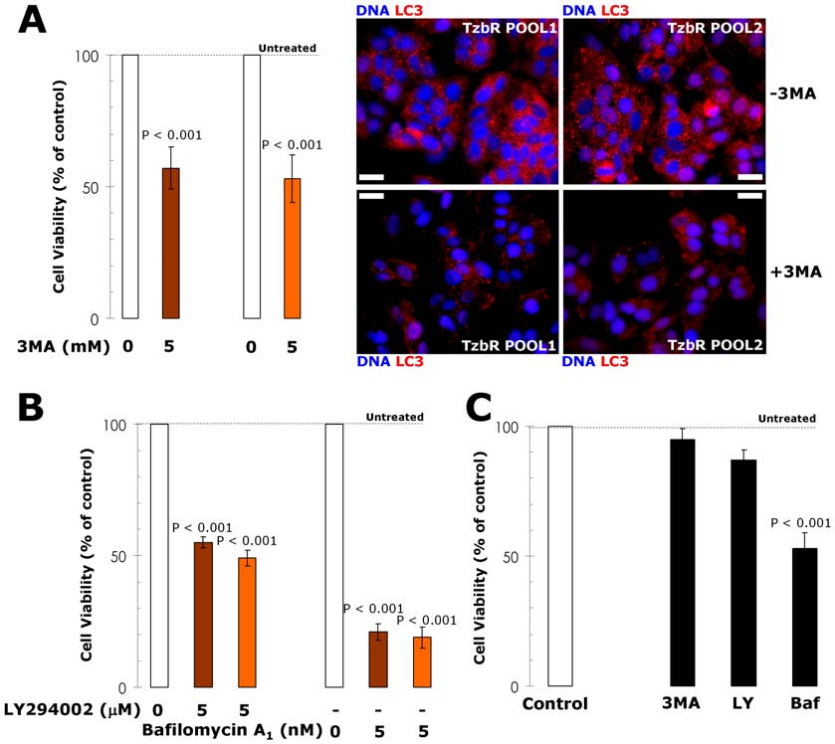
Changes in the Cell Viability of Tzb-refractory Cells Upon Pharmacological Modulation of Autophagosomes Formation/Function. The metabolic status of SKBR3 parental cells and Tzb-refractory TzbR POOLs treated with autophagy inhibitors including 3-MA, LY294002 and bafilomycin A1 was evaluated using MTT-based cell viability assays and constructing dose-response graphs as % of untreated cells (dashed line = 100% cell viability). Results are means (*columns*) and 95% confidence intervals (*bars*) of three independent experiments made in triplicate. Figure panels display results obtained with representative doses of each autophagy inhibitor, as specified. Pharmacologically-induced loss of autophagosome formation/function is highly cytotoxic to TzbR cells (A, B) compared to cell viability effects in Tzb-naive SKBR3 parental cells (C). Statistically significant differences (one-factor ANOVA analysis) between experimental conditions (*i.e.* treatment with autophagy inhibitors) and unsupplemented control cells are shown. All statistical tests were two-sided. To confirm that 3-MA-induced changes in cell viability related to 3-MA-induced changes in the number of autophagosomes in TzbR cells, cellular distribution of autophagosome marker LC3 was assessed following 72 hours exposure to 5 mM 3-MA. The total number of LC3-positive autophagosomes per cell is notably decreased in 3-MA-treated TzbR cells. Images show representative portions of SKBR3 and TzbR cell cultures captured in different channels for LC3 (*red*) and Hoechst 33258 (*blue*) with a 20× objective, and merged on BD Pathway™ 855 Bioimager System using BD Attovision™ software. *Scale bar* = 25 µm.

To pharmacologically evaluate whether Tzb-refractory cells actively used increased basal autophagy to survive Tzb therapy, we finally assessed the growth inhibitory effects of autophagy inhibitors [55]. Inhibition of the formation of pre-autophagosomal structure upon treatment with 3-methyladenine (3-MA) notably reduced cell viability in Tzb-refractory TzbR POOLs but not in Tzb-naive SKBR3 parental cells (**Figure 6**). Since 3-MA treatment may induce off-target effects, we further confirmed that 3-MA treatment was efficient at reducing the number of LC3-positive autophagosomes in TzbR cells. Representative immuno-confocal images of Tzb-refractory TzbR POOLs cultured in the absence or presence of 3-MA are shown in **Figure 6**. Cultures of TzbR cells treated with 3-MA likewise showed a significant increase in the number of cells containing <20 autophagosomes per cell. These findings, altogether, strongly suggest that increased macroautophagy actively provides a survival function to Tzb-refractory cells. This notion was further supported when similar studies were carried out in the presence of 2-(4-morpholinyl)-8-phenylchromone (LY294002). In terms of cell viability, Tzb-refractory cells were exquisitely sensitive to this agent that blocks phosphatidylinositol 3-kinase activity and prevents autophagic sequestration when compared to SKBR3 parental cells (**Figure 6**). Bafilomycin A1-prevented fusion of the autophagosomes and lysosome, and was highly cytotoxic to TzbR cells. Although less markedly, cell viability of Tzb-naive SKBR3 parental cells was also significantly reduced in the presence of bafilomycin A1 (**Figure 6**).

**Figure 7:**
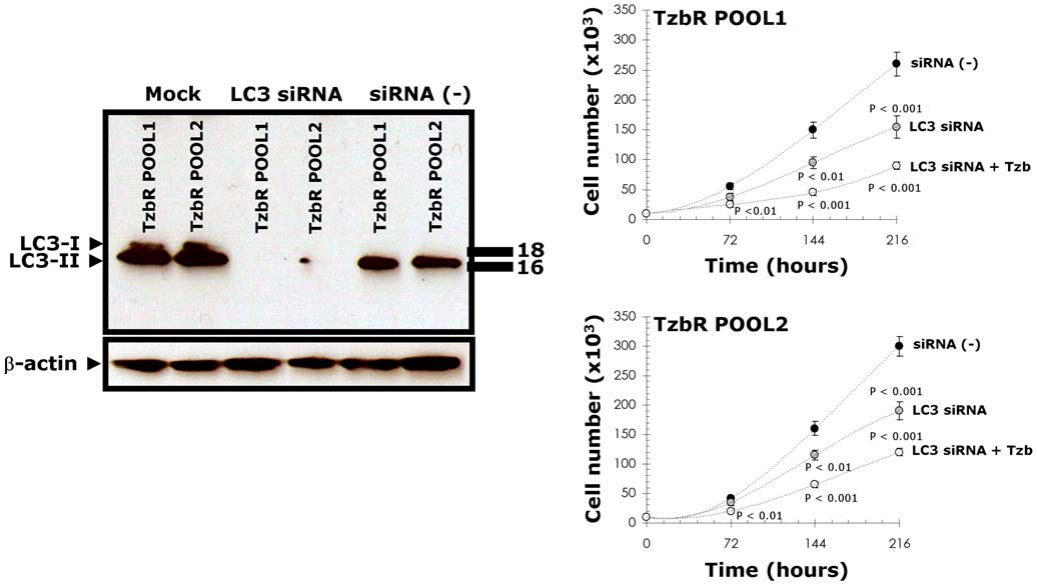
Changes in the Cell Proliferation of Tzb-refractory Cells Upon siRNA-induced Knock Down of the Autophagosome Marker LC3. Tzb-refractory TzbR POOLs were mock transfected, transfected with a non-specific control siRNA Pool (negative control) or transfected with siRNA-targeting LC3. 72 h after transfection, one set of mock-, non-specific negative control-, and RNAi LC3-transfected cells were used for immunoblotting analyses of LC3 expression. A second set of cells were harvested, re-cultured in 24-well plates at a density of 10,000 cells/well and treated with regular medium in the absence or presence of Tzb (1 µg/mL Tzb in SKBR3 parental cells and 10 µg/mL Tzb in TzbR POOLs). There were no significant changes in cell numbers when TzbR cells were treated with Tzb as single agent (omitted). siRNA-induced blockade of LC3 expression significantly reduces cell proliferation rates in TzbR POOLs. More importantly, supra-additive growth inhibitory interactions occur in LC3-depleted Tzb-treated TzbR POOLs. The data presented are mean of number cells ×10^5^/well (*circles*) and 95% confidence intervals (*bars*) of three independent experiments made in duplicate after 3, 6 and 9 days. Statistically significant differences (one-factor ANOVA analysis) between experimental conditions (*i.e.* LC3 siRNA±Tzb) and control cells (*i.e.* siRNA [-]) are shown. All statistical tests were two-sided.

To provide additional evidence that autophagy plays a critical survival role in enabling Tzb-insensitive high-rates of cell proliferation in Tzb-refractory cells and to avoid any off-target side effects that may confound interpretation of the results obtained with autophagy inhibitors, we used the potent and highly sequence-specific mechanism of RNA interference (RNAi) to block LC3-dependent autophagosome formation. Tzb-refractory TzbR POOLs transiently transfected with sequence-specific double-stranded RNA oligonucleotides targeting *Atg8/LC3* gene demonstrated hypersensitivity to high-dose Tzb. Following sequential exposure to LC3 siRNA and 200 µg/ml Tzb, TzbR cells were extremely fragile and many of the cells died before the 216-hours harvest (data not shown), thus precluding analyses of the nature of interaction. Interestingly, supra-additive (synergistic) growth inhibitory interactions occurred at late time points (*i.e.* 144 and 216 hours) when RNAi-induced knock-down of LC3 was followed by exposure to 10 µg/ml Tzb, an ineffective low-dose of Tzb when used as single agent in TzbR cells (**Figure 7**). These findings, altogether, clearly establish that hyperactivation of basal autophagy plays an essential survival role in Tzb-refractory TzbR cells re-challenged with Tzb.

### Activation of Autophagy Might Protect HER2-overexpressing Breast Cancer Cells Against Tzb

**Figure 8:**
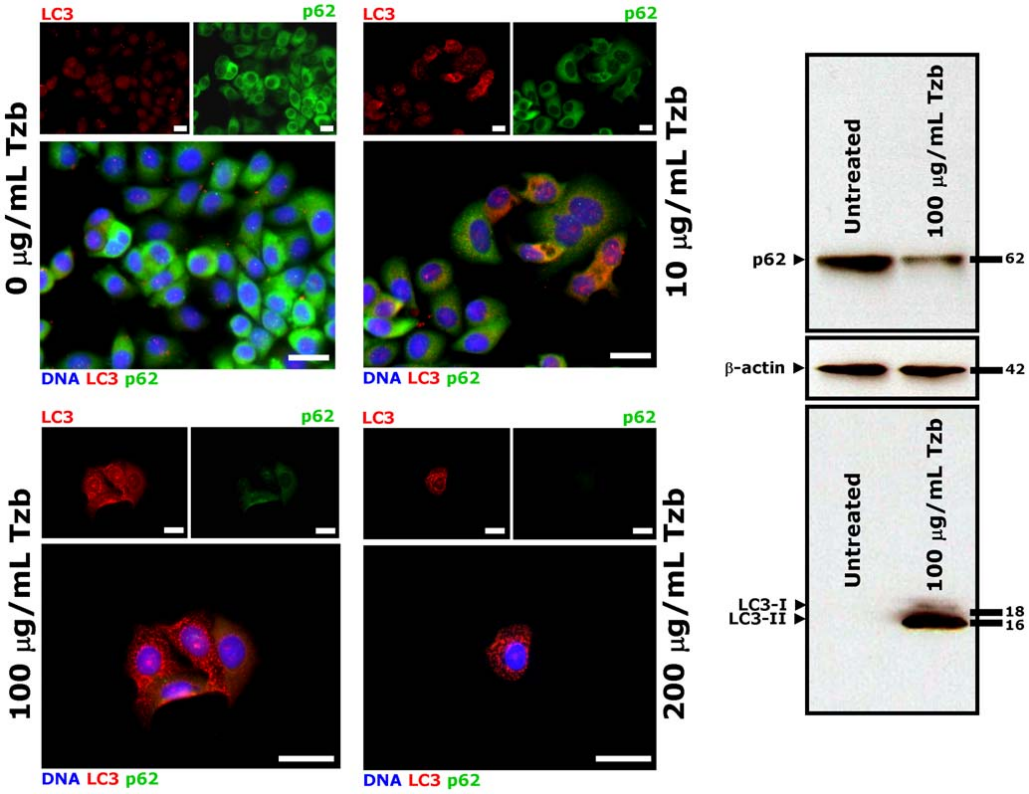
Dynamics of Autophagosome Formation and Autophagic Flux in Tzb-naive SKBR3 Cells Treated with Tzb. Tzb-naive SKBR3 parental cells were exposed to graded concentrations of Tzb (0, 10, 100 and 200 µg/mL Tzb) for 72 hours. *Immunofluorescence.* After fixation and permeabilization, cells were triple stained with LC3 and p62 antibodies and counterstained with Hoechst 33342 to visualize cell nuclei. Untreated control SKBR3 cells likewise show homogenous cytoplasmatic staining of LC3 and p62 (*i.e.* absent or low-level autophagosome formation). Surviving SKBR3 cells following exposure to graded concentrations of Tzb notably exhibit a marked contrast enhancement in the punctated pattern of LC3 (*i.e.* autophagosome formation) concurrently accompanied by p62 down-regulation (*i.e.* enhanced autophagic flux). Images show representative portions of SKBR3 and TzbR cell cultures captured in different channels for LC3 (*red*) and Hoechst 33258 (*blue*) with a 20× objective, and merged on BD Pathway™ 855 Bioimager System using BD Attovision™ software. *Scale bar* = 25 µm. *Immunoblotting.* Autophagosome formation and autophagic degradation in whole cell lysates of Tzb-treated Tzb-naive SKBR3 cells was confirmed with Western blot analyses using LC3 and p62 antibodies. High-dose Tzb notably increases LC3-II while reducing p62 expression, thus revealing an enhanced autophagic flux in the surviving fraction of Tzb-treated SKBR3 cells. Figure shows a representative immunoblotting analysis. Equivalent results were obtained in three independent experiments.

We finally speculated that macroautophagosome formation/function may contribute to cell survival of Tzb-naive HER2-positive breast cancer cells challenged to Tzb at first, thus limiting its activity and promoting further resistance. Of note, few groups of SKBR3 parental cells were found to accumulate some delicate LC3 punctate structures following treatment with low-dose Tzb (1 µg/ml Tzb). This increase in the number of autophagosomes per cell was significantly higher in isolated “SKBR3 clones” capable to survive 72 hours treatment with high-dose (100 and 200 µg/ml) Tzb (**Figure 8**). In line with this, we found that p62 expression was concomitantly decreased after Tzb treatment in this small fraction of SKBR3 cells. Tzb-promoted dynamics of autophagosomal formation (*i.e.* punctate pattern of LC3) and increased autophagic flux (*i.e.* p62 degradation) were confirmed with Western blot analyses (**Figure 8**). Similar to our findings when employing SKBR3 cells, autophagic activity was significantly elevated in Tzb-naive HER2-dependent BT474 breast carcinoma cells, whereas Tzb treatment failed to modulate autophagosome-related LC3 expression, lysosomal function and p62 expression in HER2-negative MCF-7 cells, Together, these data point to HER2 as required element in the cascade of events leading to autophagy following exposure to the anti-HER2 monoclonal antibody Tzb. As previously noted in Tzb-refractory pools, the nuclei of the adherent, autophagic surviving SKBR3 parental cells seemed viable. Although we did not evaluate if the detached cells treated with Tzb showed irregular chromatin condensation in the nucleus –which is typical of cells dying by APCD (ACDII)-, the fact that Tzb-induced macroautophagy was concomitant with a reduction in SKBR3 cell number provides evidence that Tzb-induced cell death is not an obligatory outcome of Tzb-induced macroautophagy. Therefore, macroautophagy appears to facilitate survival in Tzb-naive HER2-overexpressing human breast cancer cells, likely promoting their own resistance if Tzb-induced HER2 blockade persists.

## Discussion

We here report for the first time that the induction of autophagy is closely related to the cell survival system triggered by *HER2*-gene amplified human breast cancer cells in response to the anti-HER2 monoclonal antibody Tzb. Because a key point that needs to be emphasized when monitoring macroautophagy is that there is a difference between measurements that examine the numbers of autophagosomes *versus* those that measure flux through the autophagy pathway, we followed the “*Guidelines for the use and interpretation of assays for monitoring autophagy in higher eukaryotes*” recently presented by Klionsky and colleagues [45]. Thus, to unambiguously demonstrate that enhanced basal autophagy causally functioned to protect HER2-dependent human breast cancer cells from cell death upon chronic exposure to Tzb, we firstly measured autophagosome accumulation by fluorescence microscopy of endogenous LC3 (*i.e*. increase in punctate LC3 -a hallmark of autophagy because it is the first protein identified on the autophagosomal membrane-) and LC3-II immunoblotting (*i.e.* increase in the amount of the lipidation product of the autophagic conversion of LC3) [45]–[49]. Autophagy flux was confirmed by fluorescence microscopy and immunoblotting of p62/sequestosome-1 protein, which serves as a link between LC3 and ubiquitinated substrates destined for autophagic degradation [45], [50]–[54]. To determine that autophagy induced by Tzb provided an indispensable role in cell survival and facilitated the development of acquired resistance to Tzb, we pharmacologically impaired formation/function of macroautophagosomes by using small-molecule autophagy inhibitors. Because it is now known that pharmacological inhibitors of autophagy are not completely specific to the autophagic process and thus may produce secondary or off-target effects [55], we finally included the use of RNAi technology for specific inhibition of autophagy. These combined studies not only demonstrated that activation of basal autophagy was causally related to the acquisition of Tzb resistance but further confirmed an active role of “protective autophagy” in the maintenance of Tzb insensitivity.

The typical punctate staining that accompanies the translocation of LC3 II from the cytosol to the autophagosome membrane was detected at high levels detected in Tzb-refractory TzbR cells. Formation of autophagosomes was further enhanced in the presence of Tzb, thus suggesting that Tzb-refractory TzbR cells are uniquely characterized by their ability to sustain high levels of Tzb-induced macroautophagy without induction of cell death. Because the polyubiquitin-binding protein p62/sequestosome-1 recognizes long-lived ubiquitinated protein bodies and presents these to the autophagic machinery *via* LC3 (*i.e.* a large fraction of p62-formed protein aggregates are degraded by autophagy [50]–[54]), the fact that p62/SQSTM1 protein expression was reduced in Tzb-refractory cells supported the notion that the catabolic function of activated basal autophagy was playing a pro-survival role in Tzb-refractory cells. Since p62 downregulation was maintained in Tzb-refractory HER2-overexpressing cells chronically exposed to Tzb (*i.e.* twice weakly for a minimum of 5 months), it could be argued that reduced levels of p62/SQSTM1 may arise from reduced gene transcription or reduced translation of this protein, rather than from autophagic degradation [45]. However, p62 expression has been shown also to remain significantly decreased for several months in the chronic autophagic maladaptive response that accompanies premature aging in *Zmpste24^(−/−)^* mice [55], [56]. Moreover, immunofluorescence microscopy analyses indicated that p62 downregulation was concomitant with reduced immunoreactive for ubiquitin-positive bodies (data not shown), further suggesting that Tzb-refractory cells exhibit a significantly enhanced turnover of autophagic substrates [57], including potentially toxic aggregate-prone ubiquitinated proteins. This Tzb-resistance phenotype consistent with a chronic increase in flux through autophagic clearance pathways is in marked contrast with a newly discovered role for LC3 in nonautophagic cytoplasmic vacuolation death of cancer cells [58]. In this latter scenario, up-regulation and processing of the autophagic marker LC3 is accompanied by a marked increase in p62/SQSTM1 expression and dilation of endoplasmic reticulum due to accumulation of ubiquitinated proteins. Both chemical and genetic inhibition of autophagy demonstrated that development of acquired resistance to Tzb was due, at least in part, to activation of Autophagy in HER2-overexpressing breast cancer cells chronically cultured in the presence of Tzb. Three chemicals (3-MA, LY294002 and bafilomycin A1), which are routinely used to inhibit autophagy at different stages of the autophagosome maturation [59], significantly reduced cell viability in TzbR POOLs as measured by MTT assays. Besides the chemical autophagy inhibitors, knockdown of the autophagosome membrane protein LC3 by siRNA similarly decreased TzbR cell viability as measured by cell numbers. In contrast to either agent alone, Tzb and autophagy *LC3* gene-siRNA showed a profound combinatorial (supra-additive) effect, greatly increasing the growth inhibitory properties of Tzb. In this regard, it was noteworthy that Tzb treatment significantly increased autophagosome levels in the cytosol of viable Tzb-naive SKBR3 parental cells, thus revealing that ACDII is not an obligatory outcome of Tzb-induced macroautophagy. While further studies are clearly required to definitely uncover the ultimate molecular pathways involved (*e.g.* it could be relevant to address whether Tzb-induced non viable cells show higher levels of autophagosomes), our results suggest that autophagy may represent a general mechanism responsible for circumventing and/or delaying Tzb-induced cell death. If the magnitude of autophagy may determine the fate of Tzb-treated HER2-positive breast cancer cells (*i.e.* macroautophagy is fully induced as a survival mode but fail to rescue Tzb-sensitive cells), the ability of HER2-positive breast cancer to switch Tzb-induced autophagy to a mechanism of tumor cell survival may further explain the degree of primary (inherent) resistance to Tzb. Thresholds may exist within HER2-positive cancer cells for the magnitude of Tzb-induced autophagy that leads to cell death *versus* the magnitude of Tzb-induced autophagy that leads to cell survival. This notion would support recent findings indicating that primary Tzb resistance is not synonymous with lack of responsiveness to Tzb [60].

Autophagy is known to display a dual contrasting function in cancer cell biology. On the one hand, autophagy is a tumor suppressor mechanism and, therefore, the activation of autophagy could reverse the neoplastic phenotype. On the other hand, autophagy may contribute to tumor progression as a protective mechanism against stressful microenvironmental conditions including anti-cancer therapies [44]. From a clinical perspective, this debate is crucial in order to preferentially promote the development of therapeutic interventions that can either inhibit or enhance autophagy in tumor cells. In the cellular response to cancer therapy, a number of clinically available cancer therapeutics and experimental anticancer treatment modalities, including DNA-damaging chemotherapeutics, endocrine therapies (*e.g.* tamoxifen) and radiation therapy have been found to induce autophagy in cell culture and animal models [61]–[67]. Recent investigations have found also the presence of autophagic structures in response to molecular cancer therapies such as the TKI imatinib mesylate (Gleevec®) -the first approved drug to directly turn off the signal of a protein known to cause a cancer- and autophagy/autophagic cell death have been suggested as next targets for elimination of the resistance to imatinib in chronic myelogenous leukaemia (CML) and gastrointestinal stromal tumors (GIST) [68]–[72]. For instance, imatinib-resistant cell lines undergo cell death when concurrently treated with imatinib and the autophagy inhibitor chloroquine. Further expanding the cytoprotective role of autophagy following exposure to anti-cancer therapies, we now add the anti-HER2 monoclonal antibody as a novel molecularly targeted therapy that can trigger a pro-survival function of autophagy in HER2-dependent human breast carcinoma cells. Under experimental conditions described here, increased basal autophagy related to an increased proliferative capacity of Tzb-refractory cells translated into a significant decrease in the doubling time of Tzb-refractory cells, whereas inhibition of autophagy accelerated Tzb-induced cell death. Because recent studies have uncovered significant interactions between autophagic, apoptotic and proliferative signaling pathways [73], [74], a potential contribution of enhanced autophagic activity to efficiently maintain energy homeostasis and confer a selective growth advantage under stress conditions imposed by molecularly targeted therapies such as the anti-HER2 monoclonal antibody Tzb strongly suggests that specific inhibition of autophagic machinery may have a therapeutic role not only in HER2-positive breast cancer patients with advanced disease refractory to Tzb but also in the prevention or delay of Tzb resistance in early HER2-positive breast cancer disease.

In summary, we provide compelling data that increased autophagosome formation and function (*i.e.* enhanced autophagic flux) induced by Tzb treatment plays a critical role in HER2-positive breast cancer cell survival. HER2-overexpressing breast cancer cells chronically exposed to Tzb exhibit a *bona fide* up-regulation of the autophagic activity that efficiently works to protect themselves from the growth-inhibitory effects of Tzb. Our working model is that macroautophagosome formation and catabolic function contributes to HER2-dependent breast cancer survival and facilitates a rapid development of Tzb resistance, whereas blockade of autophagosome formation/function significantly helps to enhance the growth inhibitory activity of Tzb toward Tzb-refractory breast cancer cells. To our knowledge, these are the first examples demonstrating a synergistic nature of combining Tzb with autophagy inhibition, thus highlighting the importance of investigating autophagy knock-down as a novel means to sensitize Tzb-resistant HER2-positive breast carcinomas to the growth inhibitory actions of Tzb. Chloroquine, a drug initially developed for the treatment of malaria in the 1930s, and recently tested as autophagy inhibitor in experimental models [75]–[77], may be used in future clinical trials in combination with Tzb, which should help clarify the importance of manipulating autophagy for enhancing the therapeutic benefit of Tzb in HER2-dependent breast carcinomas.

## Materials and Methods

### Materials

Trastuzumab (Tzb; Herceptin®) -kindly provided by Hospital Universitari de Girona Dr. Josep Trueta Pharmacy (Girona, Spain)- was solubilized in bacteriostatic water for injection (USP, a sterile, nonpyrogenic preparation of water for injection containing 1.1%–1.1 mg/mL- of benzyl alcohol added as a bacteriostatic preservative)-, stored at 4°C (stock solution at 21 mg/mL) and used within one month. 3-methyladenine (3-MA) was purchased from Sigma-Chemicals (St. Louis, MO, USA) and solubilized (stock solution at 1 M) in phosphate buffered saline (PBS). Bafilomycin A1 and LY294002 were purchased from Sigma-Chemicals (St. Louis, MO, USA) and Cell Signaling Technology, respectively, and reconstituted in dimethyl sulphoxide (DMSO; stock solution at 1 mM). Rabbit anti-light-chain 3 (LC3) polyclonal antibody was purchased from MBL International Corporation (Woburn, MA, USA; PD014). Mouse anti-SQSTM1/p62 monoclonal antibody was purchased from Abcam plc. (Cambridge, UK; ab56416).

### Culture Conditions

SKBR3 breast cancer cells were obtained from the American Type Culture Collection (ATCC) and were routinely grown in Improved MEM (IMEM; Biosource International; Invitrogen S.A., Barcelona, Spain) supplemented with 10% fetal bovine serum (FBS) and 2 mM L-glutamine. Cells were maintained at 37°C in a humidified atmosphere of 95% air and 5% CO_2_. Cells were screened periodically for *Mycoplasma* contamination. For experimental use Tzb, 3-MA, bafilomycin A1 and LY294002 were prepared freshly from stock solutions and diluted with growth medium. Control cells were cultured in medium containing the same concentration (*v/v*) as the experimental cultures with treatments. The vehicle solutions had no noticeable influence on the proliferation of experimental cells.

### Establishment of Tzb-acquired Auto-resistance in HER2-positive SKBR3 breast Cancer Cells

To establish SKBR3/TzbR pools exhibiting secondary resistance to the anti-HER2 monoclonal antibody Tzb, Tzb-naive SKBR3 parental cells were exposed to increasing concentrations of Tzb for a minimum of 10 months. Briefly, SKBR3 cells were initially exposed to 20 µg/mL Tzb for 3 months (4 treatments weekly) followed by 185 µg/mL Tzb for 2 months (twice weekly). Two pools selected for further study (*i.e.* TzbR POOL1 and TzbR POOL2), resisted continuous growth in 200 µg/mL Tzb (cells were passaged at 70% confluence and Tzb-containing medium was replaced twice weakly for a minimum of 5 months). The resistant pools were maintained in medium without Tzb for at least 2 days before each experiment.

### Metabolic Status Assessment (MTT-based Cell Viability Assays)

Cell viability was determined using a standard colorimetric MTT (3-4,5-dimethylthiazol-2-yl-2, 5-diphenyl-tetrazolium bromide) reduction assay. Cells in exponential growth were harvested by trypsinization and seeded at a concentration of ∼2.5×10^3^ cells/200 µL/well into 96-well plates, and allowed an overnight period for attachment. Then the medium was removed and fresh medium along with Tzb, 3-MA, LY294002, or bafilomycin A1 was added to cultures, as specified. Control cells without agents were cultured in parallel using the same conditions with comparable media changes. Agents were not renewed during the entire period of cell exposure. Following treatment (4–5 days), the medium was removed and replaced by fresh drug-free medium (100 µL/well), and MTT (5 mg/mL in PBS) was added to each well at a 1/10 volume. After incubation for 2–3 hr at 37°C, the supernatants were carefully aspirated, 100 µL of DMSO were added to each well, and the plates agitated to dissolve the crystal product. Optical density (OD) was measured at 570 nm using a multi-well plate reader (Model Anthos Labtec 2010 1.7 reader). Cell viability after exposure of cells to drugs was analyzed as percentages of the control cell absorbances, which were obtained from control wells treated with appropriate concentrations of the agents' vehicles that were processed simultaneously. For each treatment, cell viability was evaluated as a percentage using the following equation: (OD_570_ of treated sample/OD_570_ of untreated sample) ×100.

### Transient transfection of siRNAs

The siRNA sequences used for targeted silencing of human LC3 (MAP LC3β siRNA (h): sc-43390) were supplied by Santa Cruz Biotechnology (Santa Cruz, CA, USA) as ready-to-use pools of 3 to 5 target-specific 19–25 nt double-stranded siRNAs designed to efficiently knock down the expression of LC3 gene. siRNA A (sc-37007), which consists of a scrambled sequence that will not lead to the specific degradation of any known cellular mRNA, was employed as negative control for experiments using LC3-targeted siRNA transfection. Transfections were performed as described in Santa Cruz technical bulletin. Briefly, cells at a confluence of 60 to 80% were transfected with the selected siRNAs using Santa Cruz Biotechnology's siRNA Transfection Reagent (sc-29528) and siRNA Transfection Medium (sc-36868) following the manufacturer's instructions.

### Cell proliferation assays

Cells were trypsinized and re-plated in 24-well plates at a density of 10,000 cells/well. Cells were incubated for 18 h to allow for attachment, after which a zero time point was determined. Cells were then cultured in regular medium containing 5% FBS) in the absence or presence of Tzb, and counted at days 0, 3, 6 and 9 with a Coulter Counter (Coulter Electronics, Inc., Hialeah, FL, USA). All assays were performed at least three times in duplicate.

### Immunofluorescence staining and high-content confocal imaging

Cells were seeded at approximately 5,000 cells/well in 96-well clear bottom imaging tissue culture plates (Becton Dickinson Biosciences; San Jose, CA, USA) optimized for automated imaging applications. Triton® X-100 permeabilization and blocking, primary antibody staining (1∶50 dilution), secondary antibody staining using Alexa Fluor® 488/594 goat anti-rabbit/mouse IgGs (Invitrogen, Molecular Probes, Eugene, Oregon, USA) and counterstaining (using Hoechst 33258; Invitrogen) were performed following BD Biosciences protocols. Images were captured in different channels for Alexa Fluor® 488 (pseudo-colored green), Alexa Fluor® 594 (pseudo-colored red) and Hoechst 33258 (pseudo-colored blue) on a BD Pathway™ 855 Bioimager System (Becton Dickinson Biosciences, San Jose, California, USA) with 20× or 40× objectives (NA 075 Olympus). Merged images, confocal Z stack acquisition and 3D visualization were obtained according to the Recommended Assay Procedure using BD Attovision™ software.

### Immunoblotting procedures

Cells were washed twice with cold-PBS and then lysed in buffer (20 mM Tris pH 7.5, 150 mM NaCl, 1 mM EDTA, 1 mM EGTA, 1% Triton® X-100, 2.5 mM sodium pyrophosphate, 1 mM β-glycerolphosphate, 1 mM Na_3_VO_4_, 1 µg/mL leupeptin, 1 mM phenylmethylsulfonylfluoride, and complete protease inhibitor cocktail [Sigma-Chemicals; St. Louis, MO, USA]) for 30 minutes on ice. The lysates were cleared by centrifugation in an Eppendorff tube (15 minutes at 14,000×g, 4°C). Protein content was determined against a standardized control using the Pierce Protein Assay Kit (Rockford, IL, USA). Equal amounts of protein (*i.e.* 50 µg) were resuspended in 5× Laemmli sample buffer (10 min at 70°C), resolved by electrophoresis on 10% SDS-PAGE, and transferred onto nitrocellulose membranes. Non-specific binding on the nitrocellulose filter paper was minimized by blocking for 1 h at RT with TBS-T buffer [25 mM Tris-HCl (pH 7.5), 150 mM NaCl, 0.05% Tween 20] containing 5% (*w/v*) nonfat dry milk. The treated filters were washed in TBS-T and then incubated with anti-LC3 (1∶1000 dilution) or anti-p62 (1∶1000 dilution) antibodies, as specified, in 5% *w/v* BSA, 1× TBS-T buffer, 0.1% Tween-20 at 4°C with gentle shaking, overnight. The membranes were washed in TBS-T, horseradish peroxidase-conjugated secondary anti-mouse/rabbit IgGs in TBS-T was added for 1 h, and immunoreactive bands were detected by chemiluminiscence reagent (Pierce, Rockford, IL, USA). Experiments involving immunoblotting procedures were repeated at least three times and blots were re-probed with an antibody for β-actin to control for protein loading and transfer. Densitometric values of proteins bands were quantified using the Scion Image software (Scion Corporation, Frederick, MD, USA).

### Statistics

Two-group comparisons were performed by the Student *t* test for paired and unpaired values. Comparisons of means of ≥3 groups were performed by ANOVA, and the existence of individual differences, in case of significant *F* values at ANOVA, tested by Scheffé's multiple comparisons.

## Supporting Information

Video S1 (0.16 MB WMV)

Video S2 (0.63 MB WMV)

Video S3 (0.65 MB WMV)
